# Population-based Estimates of Acute Gastrointestinal and Foodborne Illness in Barbados: A Retrospective Cross-sectional Study

**Published:** 2013-12

**Authors:** Maria Ingram, Joy St. John, Tyrone Applewhaite, Pamela Gaskin, Karen Springer, Lisa Indar

**Affiliations:** ^1^Ministry of Health, Barbados; ^2^University of the West Indies, Cave Hill Campus, Barbados; ^3^Caribbean Epidemiology Centre (CAREC/PAHO/WHO), Trinidad and Tobago

**Keywords:** Foodborne illness, Morbidity, Population survey, Prevalence, Barbados

## Abstract

The aim of this study was to determine the burden and impact of acute gastroenteritis (AGE) and foodborne diseases (FBDs) in Barbados through a retrospective, cross-sectional population survey and laboratory study in August 2010–August 2011. Face-to-face interviews were conducted with one person from each of 1,710 randomly-selected households. Of these, 1,433 (84%) interviews were completed. A total of 70 respondents reported having experienced AGE in the 28 days prior to the interview, representing a prevalence of 4.9% and an annual incidence rate of 0.652 episodes per person-year. Age (p=0.01132), season (p=0.00343), and income (p<0.005) were statistically associated with the occurrence of AGE in the population. Norovirus was the leading foodborne pathogen causing AGE-related illness. An estimated 44,270 cases of AGE were found to occur during the period of the study and, for every case of AGE detected by surveillance, an additional 204 cases occurred in the community. Economic costs of AGE ranged from BD$ 9.5 million to 16.5 million (US$ 4.25-8.25) annually. This study demonstrated that the public-health burden and impact of AGE and FBD in Barbados were high and provided the necessary baseline information to guide targeted interventions.

## INTRODUCTION

Acute gastroenteritis (AGE) is a significant but preventable cause of morbidity and mortality worldwide. In 2005, an estimated 1.5 million people died of diarrhoeal diseases worldwide (1). The World Health Organization (WHO) and a recent study estimated that at least 70% of diarrhoeal diseases can be attributed to foodborne pathogens (2-4). This infers that at least 3,000 lives were lost daily due to foodborne diseases. In addition to the significant morbidity and mortality attributed to AGE and foodborne diseases (FBD), estimates from developed nations, such as the United States, have reported huge financial costs (3,5). These costs further emphasize the significant public-health burden of these conditions (6,7).

Although numerous studies aimed at estimating the burden of AGE and FBDs have been conducted, differences in the surveillance systems of countries and the methodologies used prevented valid comparisons (3). Consequently, the global burden and impact of acute gastrointestinal illness and foodborne diseases are unknown (8,9).

In response, the WHO, through the Global Burden of Disease Initiative, developed a methodology for the estimation of the burden of AGE and FBDs (10,11). The aim of the Burden of Illness (BOI) studies is to estimate the true number of cases of a disease in the population, and this requires quantifying the prevalence or incidence of the disease in the population and determining underreporting and underdiagnoses at different levels of the reporting system. A BOI reporting pyramid is used as a model for understanding AGE and FBD reporting (8). Notably, this pyramid mirrors empirical evidence, which demonstrates that the majority of illnesses in a community (at the base of the pyramid) are not reported to the national authorities (peak of the pyramid).

The Caribbean is one of the most tourist-dependent regions in the world and, as such, a critical region for which the WHO has little information on the epidemiology of acute gastrointestinal illness and foodborne diseases. Moreover, in addition to the lack of precise estimates, the Caribbean Epidemiology Centre (CAREC) reports that surveillance data for the region show high and increasing numbers of AGE (12). This trend suggests high/increasing levels of foodborne diseases in the region (12). Foodborne disease outbreaks have frequently occurred and are of great concern. Some of these outbreaks have been large, affecting hundreds of persons in the tourism sector (hotels/cruise ships) and have resulted in international concern.

CAREC is leading the burden of acute gastroenteritis and foodborne disease study in selected Caribbean countries, one of which was Barbados. This multicentred study is part of the larger WHO initiative to understand and reduce the global burden of acute gastrointestinal illness and foodborne diseases (1). The study offered a unique opportunity for Barbados to determine the public-health burden and impact of AGE and FBDs and to evaluate the sensitivity of its surveillance system. Information garnered from this study will be useful for setting priorities, developing public-health policies, implementing interventions, and allocating resources to areas of greatest concern. This study also provided valuable information that will inform and strengthen foodborne disease surveillance.

## MATERIALS AND METHODS

### Study site

Barbados is the most easterly point of the Caribbean Islands, with an area of 430 sq. km and an estimated population of 285,000 (13). The island boasts of a vibrant tourism industry and is one of the most tourism-dependent islands of the Caribbean (14). In Barbados, communicable disease surveillance is conducted both passively and actively, and like most other systems of this type, significant underreporting occurs. This is especially so for the more prevalent conditions, such as AGE and dengue fever. Based on syndromic surveillance, the number of AGE cases reported to the surveillance unit was 1,847 and 1,859 in 2008 and 2009 respectively. These cases represent 35% of the syndromes reported and an average annual incidence of 650 cases of AGE per 100,000 population.

### Study design

The overall study is composed of two interlinked cross-sectional surveys—a retrospective, cross-sectional population-based survey and a laboratory-based survey. Both of these surveys were conducted during August 2010–August 2011. The population survey was conducted in two phases. According to surveillance data, the low-AGE season spans the months of May-January whereas the high-AGE season spans the months of February-April. Phase 1 of the study was conducted during the low-AGE season (August and September) and Phase 2 of the study was conducted in the high season (March-April).

### Sample-size and data collection

The required sample-size of 1,433 for the population study was determined using an assumed AGE prevalence of 40% (L. Indar. Personal Communication, 2010) based on internal estimates from CAREC, an allowable error of 3%, and a design effect of 1.4 at 95% confidence interval. The adjusted sample-size (non-response rate of 20%) was 1,719. The final sample-size used was 1,710. All samples received by the Laboratory were used for analysis in the laboratory survey. For the population-based study using multistage sampling, the 11 parishes of Barbados were further divided into 583 localities (sampling frame) (15). These localities were stratified using probability proportional-to-size. Within each stratum, localities (primary sampling units) were randomly selected. From each locality, households were systematically chosen; and from each household, the person with the next birthday falling before the day of the interview was recruited into the study. If there was more than one eligible person in the household, the person to be interviewed was randomly selected using a fair coin. If the eligible person was not available at the time of a visit, the interviewer scheduled a time when the person would be available and returned to the house for a second time. Households were visited for a maximum of 3 times.

A structured standardized questionnaire, developed by the Caribbean BOI team, was used for collecting data. This questionnaire was administered by face-to-face interviews by trained university and college students. Information on the presence or absence of diarrhoea in the 28 days prior to the interview, secondary symptoms of cases, demographic and socioeconomic characteristics, such as age, gender, education, total household income, the number of individuals residing in the household, and healthcare-seeking behaviour was collected. Information on risky practices, such as infrequent handwashing and consumption of raw or undercooked foods was also collected. Interviews were conducted in two phases. Eight hundred and fifty-five interviews were conducted in each phase.

### Inclusion and exclusion criteria

Acute gastrointestinal illness (AGI) was defined as acute (sudden) onset of diarrhoea (3 or more loose or watery stools in the past 24 hours), with or without fever (>38 °C) and presenting with or without dehydration, vomiting, and/or visible blood. Eligible participants were Barbadian residents aged 1 year or older.

Eligible participants unwilling or unable to participate, vulnerable population groups, children below the age of 18 years without parental consent, and persons who were diagnosed with chronic gastrointestinal illness were excluded from this study.

### Ethical considerations

The survey was pretested through previous exercises conducted in other study sites. Ethical approval was obtained from the Institutional Review Board of University of the West Indies, Ministry of Health, Barbados. Informed consent was sought from study participants. Each participant was informed of the purpose of the survey, any risks involved, and the anticipated benefits of the study. Participants were also assured that participation was voluntary and that they could withdraw from the study anytime. Participants were asked to sign a consent form before the questionnaire was administered. Anonymity was also maintained; hence, the names of the participants in this study were not collected, and each participant entering the survey was assigned a unique identification number.

### Validation, entry, and analysis of data

Quality control in this study included validation visits to the field and double entry of the questionnaire during data-entry. The biostatistical package Epi Info (version 7) was used for data-entry and analysis. Univariate analysis was done to describe the study population. Following this, bivariate analysis using the chi-square test was done to investigate differences in AGE prevalence among different age-groups and genders. Logistic regression was used in examining the relationship between the prevalence of AGE and age, gender, and household income.

### Laboratory survey

A retrospective baseline survey aimed at evaluating laboratory practices was conducted with public and private laboratories in Barbados one year prior to the study. This exercise was aimed at strengthening the capacity of the laboratories and included the identification of areas of weaknesses in the laboratory surveillance of AGE and foodborne pathogens and standardization of isolation methodologies. During the study period (1 year), the survey tool (questionnaire) collected data on laboratory procedures, turnaround times, media and supplies used, the type and annual number of tests that were conducted, and reporting system. This component of the BOI study provided an estimate of the number of specimens received in the laboratories, the proportion of cases lost to surveillance because of negative findings, and the proportion of confirmed cases reporting to the surveillance systems.

## RESULTS

### Response rate

Of the 1,710 eligible participants, 1,433 participated in the study; of the expected 855 from each phase, 695 responded in Phase 1 and 738 in Phase 2, thus yielding an overall response rate of 84%.

### Gender distribution

**Figure 1.**
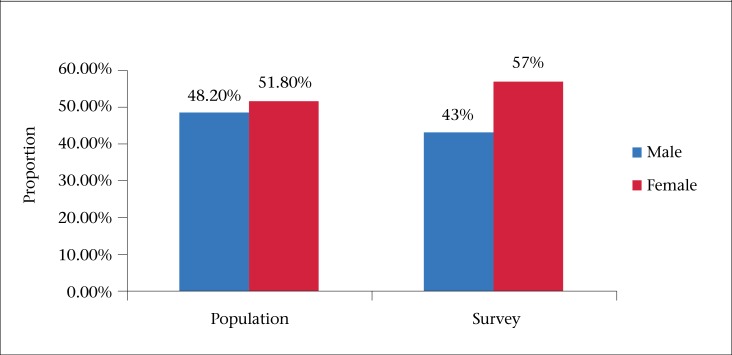
Gender distribution (proportion) in the population and survey respondents of the Burden of Illness Study, Barbados

**Figure 2.**
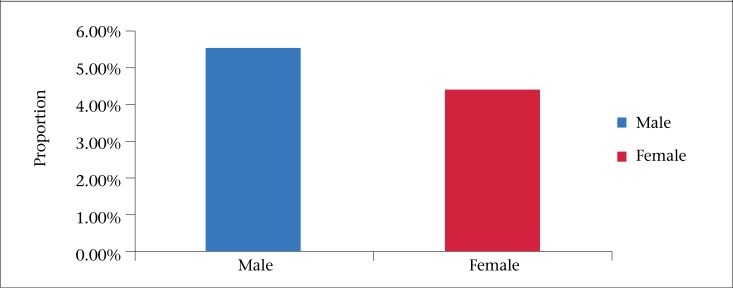
Prevalence of acute gastrointestinal illness (AGI), BOI Study, Barbados, August 2010-2011

Fifty-seven percent of the survey respondents were female. When compared with the general population distribution (Table 1 and Figure 1), females were overrepresented in this study. The prevalence of AGE was also shown to be 25% higher among males (Figure 2).

### Age distribution

**Figure 3.**
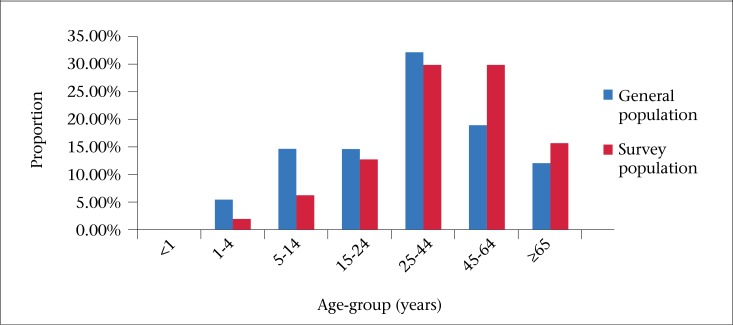
Age-group distribution in the population and the study respondents in Barbados

**Figure 4.**
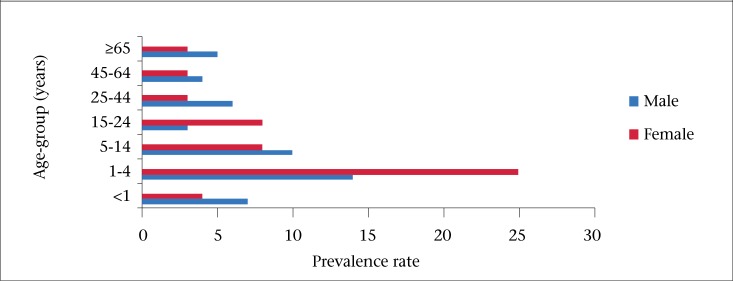
Monthly prevalence of AGE by age-group and gender, BOI Study, Barbados, August 2010–August 2011

Most (88%) of the study population was between the age of 15 and 65 years. Compared to the general population, children aged 0-14 year(s) were underrepresented (12.2%, vs 21.5%) (Table 1, Figure 3). The prevalence of AGE was the highest in the 1-4 year(s) age-group (Figure 4), and the age was shown to be a significant risk factor (p=0.011) for acute gastroin­testinal illness in this study.

### Ethnicity

Ninety-four percent of the study population was Afro-Caribbean (Table 1). Other population groups were Indian (1.2%), Asian (0.2%), European (1.0%), South American (0.3%), and North American (2%).

### Household income

Sixty-one percent (872/1,433) of the study respondents provided information on their household income. Of those who gave a response to this query, 45% were from households regarded as low-income, 42% represented middle-income and 13% represented high-income households. In this study, acute gastrointestinal illness was shown to be significantly associated with income (p=0.005) (Table 1). The odds of AGE in the high-income household category were twice (OR 2.4) that of the reference group (low-income category).

**Table 1.** Demographic characteristics of residents, survey respondents, and description of the monthly prevalence of self-reported acute gastrointestinal illness per category

Variable	Residents (N=268,792)	Respondents	Prevalence of AGE	95% Confidence interval
Sex (p=0.3330)		(n=1,433)		
Male	133,706 (48.2%)	616 (43%)	34 (5.52%)	3.98-7.61
Female	142,596 (51.8%)	817 (57%)	36 (4.41%)	3.20-6.04
Age (completed years) (p=0.01132)		(n=1,427)		
<1	3,469 (1.3%)	38 (2.7%)	2 (5.26%)	1.45-17.28
1-4	14,954 (5.5%)	30 (2.1%)	6 (20%)	9.51-37.31
5-14	40,210 (14.7%)	92 (6.4%)	8 (8.7%)	4.48-16.24
15-24	40,142 (14.7%)	184 (12.8%)	11 (6.0%)	3.37-10.39
25-44	88,137 (32.2%)	429 (29.9%)	20 (4.7%)	3.04-7.09
45-64	53,161 (19%)	428 (29.9%)	15 (3.5%)	2.13-5.70
≥65	33,085 (12.1%)	226 (15.8%)	8 (3.5%)	1.80-6.83
Cultural group (p=0.88157)		(n=1,432)		
African/Black		1,352 (94%)	65 (4.81%)	3.79-6.08
Indian		17 (1.2%)	1 (5.88%)	1.05-26.98
Asian		3 (0.2%)	0.0%	0.0
European		15 (1.0%)	1 (6.67%)	1.19-29.8
South American		4 (0.3%)	0.0%	0.0
North American		29 (2.0%)	1 (3.45%)	0.61-17.2
Other		12 (0.8%)	2 (16.67%)	4.7-44.8
Monthly income* (BD$) (p>0.000)		(n=872)		
Low income (0-2,500)		392 (45.0%)	20 (5.10%)	3.33-7.75
Medium income (2,501-6,000)		367 (42.1%)	17 (4.63%)	2.91-7.29
High income (>6,000)		113 (12.9%)	13 (1.15%)	6.84-18.69
Education (Mother) (p=0.92712)		(n=1, 123)		
Primary		141 (12.6%)	7 (4.96%)	2.42-9.89
Secondary		506 (45.1%)	26 (5.14%)	3.53-7.42
Certificate/Diploma		251 (22.3%)	15 (5.98%)	3.66-9.63
Undergraduate/Graduate		152 (13.5%)	6 (3.95%)	1.82-8.35
Postgraduate		73 (6.5%)	5 (6.85%)	2.96-15.05
Education (Father) (p=0.69309)		(n=916)		
Primary		98 (10.7%)	4 (4.08%)	1.60-10.03
Secondary		439 (47.9%)	23 (5.24%)	3.52-7.74
Certificate/Diploma		195 (21.3%)	8 (4.1%)	2.09-7.88
Undergraduate/Graduate		105 (11.5%)	8 (7.62%)	3.91-14.32
Postgraduate		79 (8.6%)	6 (7.59%)	3.52-15.59
People in household		3 (Median)	4 (Median)	

*Changed income to 3 categories

### Education

Most male heads of the households had attained up to primary (64.7%) and secondary (19.5%) level of education; and 62.9% of female heads of households had attained primary level and 19.6% secondary level of education (Table 1).

### Magnitude of illness

Seventy-seven (5.4%) of the study population reported that they had experienced diarrhoea (3 or more watery or loose stools within 24 hours with or without fever, vomiting, or visible blood in the stool) in the four weeks prior to the interview and were, therefore, classified as self-reported cases of AGE. Six of these cases, however, stated that their symptoms were due to a chronic condition, and one person was unsure.

Since the objective of the study was to describe AGE, these 7 respondents were classified as non-cases and were excluded from analysis. Of the remaining 70 cases of AGE, 20 representing 28.6% reported more than one episode in the 28 days prior to the interview. The annual incidence rate was 0.652 episodes per person-year; the calculation is shown in the Appendix.

### Symptoms and severity

Of the 70 self-reported AGE cases occurring during the study year, the most common secondary symptoms were abdominal pain (61%), nausea (42.9%), vomiting (34.3%), and headache (32.9%). The maximum number of stools in 24-hour period ranged from 3 to 15, with a mean of 5 and a median of 5. On average, ill persons experienced an AGE-related illness for 2 days, with a range of 1-7 day(s). The majority (96%, 67/70) of cases reported restricted activity, spending at least one day at home due to their illness. Fourteen cases required other individuals to look after them while ill. The range of days taking care of a case was 1-7 day(s), with a median of 2 days. Twenty-three percent of the ill respondents reported AGE among other household members.

### Temporal and spatial distribution of AGE

Based on syndromic surveillance, the number of AGE cases reported to the surveillance unit clearly indicates distinct low- and high-AGE seasons in Barbados. Our findings showed a similar temporal distribution. Seventy percent of cases occurred in the high season, and the prevalence of AGE during this period ranged from 3.6 to 10.7 in the sentinel sites. The prevalence during the low season ranged from 0.0 to 4.7 (Table 2).

**Figure 5.**
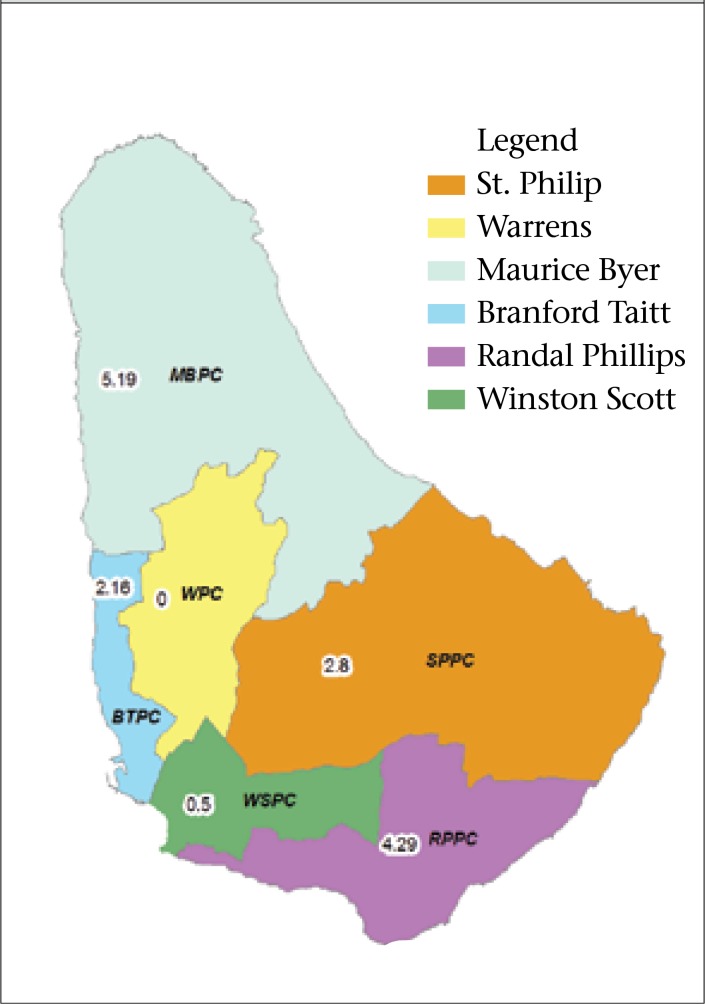
Prevalence ratio of AGE in health regions, using Warrens Health Region as the reference region, 2010-2011

Compared to the Warrens catchment area, the highest monthly prevalence of self-reported cases of AGE occurred in the Randal Phillips, Maurice Byer, and St. Phillip catchments (Figure 5). Notably included in these catchments is the tourist belt. This difference in prevalence across the health regions was statistically significant (p=0.0144). Regarding the burden that we approximate from this exercise, notably 57% suggest that our approximation may be low/underestimated.

### Healthcare-seeking behaviour/Use of medical systems

Twenty-five of the 70 cases representing 35.7% sought medical care for their illness. Eight (32%) attended a public healthcare system (polyclinic); similarly, 8 (32%) attended a private doctor or emergency clinic. One (4%) case was hospitalized, and the remaining 32% attended a private clinic. Most (18/25, 72%) of these cases reported having medication prescribed. Six were prescribed antibiotics, two of whom completed the course. Four individuals were prescribed oral rehydration solution. Sixteen cases took non-prescribed medications for their illnesses. Of them, 3 took ‘unknown bush medicine’. Four (16%) of the cases had a stool specimen requested for (Table 3).

### Risk factors, habits, and hygiene

Cases were asked to identify what factors, in their opinion, contributed or caused their illness. Thirty-one representing 44.3% stated that they believed they became ill from something they consumed whereas 13 (18.57%) believed that they contracted their illness through contact with another sick person.

**Table 2.** Prevalence of self-reported cases of AGE by health region

Health district	Low/High-AGE season	Number of respondents	Number of cases	Prevalence of AGE cases per health district (95% CI)	Prevalence ratio of cases/district
Black Rock Polyclinic	Low	74	1	1.35% (5.3-13.2)	3.04
	High	73	3	4.11% (6.0-15.7)	
Maurice Byer Polyclinic	Low	68	0	0.0%	7.32
	High	82	6	7.32% (3.40-15.06)	
Randal Phillips Polyclinic	Low	113	2	1.77% (0.49-6.22)	6.05
	High	140	15	10.7% (6.60-16.92)	
St. Philip Polyclinic	Low	50	1	2.0% (0.35-10.5)	3.95
	High	114	9	7.89% (4.21-14.32)	
Winston Scott Polyclinic	Low	252	12	4.76% (2.74-8.14)	0.75
	High	139	5	3.6% (1.55-8.15)	
Warrens Polyclinic	Low	138	6	4.35% (2.01-9.16)	1.41
	High	163	10	6.13% (3.36-10.92)	

**Table 3.** Healthcare-seeking behaviour, BOI Study, Barbados, August 2010–August 2011

Healthcare-seeking behaviour	Number	**%**	95% CI
Number of cases seeking medical care (n=70)	25	35.7	24.6-48.1
Number of cases asked to submit specimen (n=25)	4	16.0	4.54-36.08
Number submitting specimen (n=4)	2	50.0	6.76-93.24
Number of cases prescribed medication (n=25)	18	72.0	50.61-87.93
Number taking antibiotics (n=18)	6	33.0	16.3-56.25
Number taking oral rehydration fluid (n=18)	5	27.7	9.0-45.2
Number taking non-prescribed medications (n=70)	16	22.9.0	14.6-33.9
Number of stools in 24 hours	5 (Mean)	5 (Median)	3-15 (Range)

### Household-size

Respondents were asked about the number of individuals living in the household; this ranged from 1 to 11 persons, with a median of 3 persons in the household whereas the households of cases had a median of 4 persons. This difference was not statistically significant (p=0.19304). Additionally, 19 cases reported that another individual was ill with diarrhoea in their house at the same time of their illness.

### Handwashing

One out of every four persons in this study reported that they did not wash their hands with soap prior to eating.

### Contact with animals

Of the 70 self-reported cases of AGE, 22 representing 31.4% stated that they had had contact with one or more animals (Table 5).

### Consumption of high-risk foods

Of the 70 self-reported AGE cases, 3 cases representing 4.3% had consumed one or more of the high-risk foods in the month prior to the interview (Table 5).

**Table 4.** Laboratory practices by pathogen, BOI Study, Barbados, August 2010–August 2011

+Pathogen(s) tested in AGE diarrhoeal stool at laboratory	Sensitivity of test (%)*	How often does lab test for pathogen done+	Actual lab data at the lab	Number and % reported to MOH+	% reported to CAREC on four weekly forms++
*Salmonella*	98	100%	109	108 (108/109=99%)	108 (108/109=99%)
*Shigella*	98	100%	0	0	0
*Campylobacter*	98	100%	71	69 (69/71=97%)	65 (65/70=91.5%)
*E. coli* 0157:H7	95	5%	0	0	0
*S. aureus*	98	0	1	1 (1/1=100%)	0 0%
Rotavirus	95	55%	9	9 (9/9=100%)	8 (8/9=88.8%)
Norovirus	73	25% (322/1261)	26	8 (8/26=30.7%)	0 0%
Parasites (*Giardia*)	75	95%	1	0	0
Total			217	198 (91%)	181 (83%)

*Sensitivity: for culture tests, this is subjective, based on lab directors’ knowledge of lab capacity;

for test-kits, the sensitivity stated on test kit;

+Source: Ministry of Health Surveillance Unit, Barbados;

++Source: CAREC Four-weekly reports

### Purchasing risky foods

Of the 70 self-reported cases, 62/65 representing 95% purchased their chicken frozen or chilled, 5 did not purchase chicken, and 2 purchased live chicken. There was no association between purchasing chicken and AGE.

### Laboratory results

Of the 2,989 stool samples received during the period of the study, 57.3% were non-diarrhoeal. From the 1,275 diarrhoeal samples, 217 were found to be positive for a foodborne pathogen (Table 4).

### Estimating the burden of syndromic and laboratory-confirmed AGE

**Figure 6.**
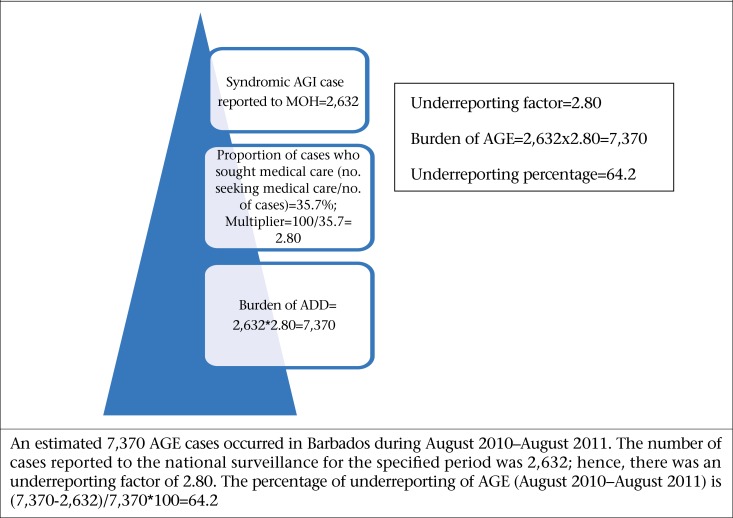
Estimated burden of AGE during August 2010–August 2011 using syndromic surveillance data

**Figure 7.**
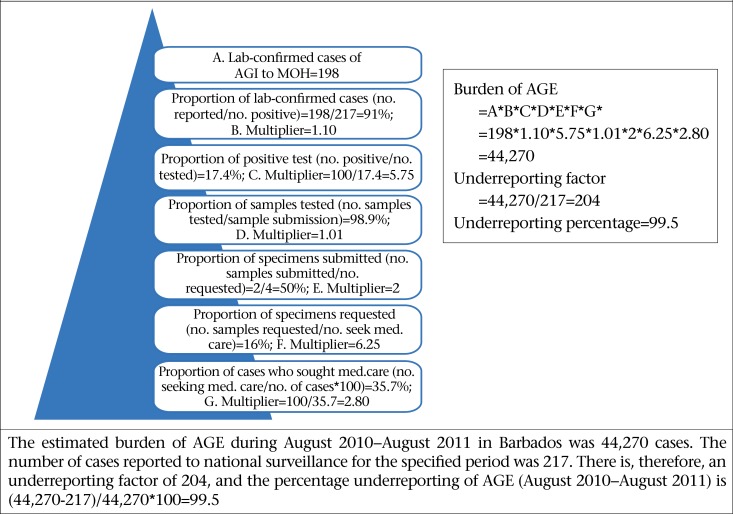
Estimated burden of laboratory-confirmed AGE for the period August 2010–August 2011

Figure 6 and 7 illustrate the calculation of precise estimates of the burden of syndromic and laboratory-confirmed AGE for the study period. An estimated 7,370 syndromic cases and an estimated 44,270 laboratory-confirmed cases occurred in this period. In contrast, 2,632 and 198 cases were reported to the Ministry of Health. These estimates, therefore, demonstrate that, for every one case of syndromic reported AGE, an additional 3 cases occurred within the population and, likewise, for every case of laboratory-confirmed FBD/AGE pathogen, 204 additional cases occurred within the population.

### Economic burden of AGE in Barbados, 2010-2011

Using cost data from the private healthcare system, a crude estimate of the economic impact of AGE was calculated. This estimate was calculated using the minimum costs for basic medical services and treatment. The estimated economic burden for AGE ranges from BD$ 9.5 million to 16.5 million (US$ 4.75-8.26 million).

## DISCUSSION

Although acute gastroenteritis (AGE) remains an important public-health issue in both developed and developing countries, few population-level studies of AGE and foodborne diseases have been conducted in developing regions (11). This study provides the first population-based estimates of the magnitude, distribution, epidemiology, and burden of AGE and foodborne diseases (FBDs) in Barbados. Key strengths of this study are the high response rate and the fact that all ages and both genders were represented. These factors contribute to the representativeness of the sample and the external validity of the results.

**Table 5.** Respondents’ exposures to animals or high-risk food items in the burden of AGE in Barbados

Route of transmission	No. of respondents	Overall monthly prevalence (n=70) No. (%)	95% CI
Contact with animals			
Sheep/goats	47	4 (5.7)	1.6-13.9
Pigs	27	1 (7.1)	0.2-33.9
Poultry	82	4 (5.7)	1.6-13.9
Dogs	538	31 (44.3)	32.4-56.7
Cats	254	10 (14.3)	7.0-24.0
No animals	304	22 (7.2)	4.7-10.9
Ate raw or undercooked foods			
Raw eggs	6	1 (1.43)	0.04-7.7
Undercooked eggs	19	0.00	0.00
Raw seafood	13	2 (2.86)	0.35-9.94
Purchased uncooked chicken			
Live	46	2 (2.9)	0.35-9.94
Frozen	715	33 (47.1)	35.1-59.5
Refrigerated	523	29 (41.4)	29.8-53.8
Did not purchase	108	5 (7.14)	2.36-15.89

Evidence of a large burden of AGE and FBD has been demonstrated with an estimated burden of 44,760 cases of laboratory-confirmed AGE, with an underreporting of 99.5% and an underreporting factor of 204. This study also revealed that acute gastrointestinal illness is responsible for at least 88,540 person-days of illness, 632 admissions to hospital, 88,540 person-days lost from work, 15,937 physician consultations, and 4,194 prescriptions among the Barbadian population each year. Using cost data from private healthcare systems, the economic burden is crudely estimated to be as much as 16.5 million Barbados dollar (US$ 8.26 million).

The magnitude of morbidity and its associated costs demonstrate that the public-health burden and impact of AGE and FBD in Barbados are high. These estimates also emphasize the enormity of the degree of underreporting and underdiagnosis of AGE and foodborne pathogens at all stages of the reporting pyramid in Barbados and mirror the results of previous BOI studies conducted in other countries, such as Cuba, Canada, and Jordan (8,16,17). Notably, a large proportion (57%) of samples submitted to the laboratory was non-diarrhoeal and, therefore, could not be evaluated. This suggests that the burden of FBD and AGE is likely to be higher than has been reported in this study.

A review of literature showed that, when compared with studies conducted in other countries, such as Cuba, Canada, and Chile, the self-reported prevalence of AGE in Barbados is markedly lower. This amounts to half of the reported prevalence in these countries (10.6%, 9.2%, and 9.2% respectively) (16). However, the annual incidence rate of 0.65 episodes per person-year compares favourably with scenarios in other countries, such as Ireland where the incidence rate is 0.6 episodes per person-year (11).

Gender disparity was observed in this study. The self-reported prevalence of AGE was found to be 25% higher among males. In contrast, higher rates in females have been observed in other studies (18-21). We postulate that gender differentials in risky behaviour and, hence, exposure to contaminated foods are likely to be the reason for the observed difference. Males in Barbados tend to eat outside the home more than females.

Age (p=0.01132), season (p=0.00343), and household income (p=0.005) were found to be statistically associated with the occurrence of AGE in the population. The odds ratio of AGE was the greatest among those below the age of 4 years compared to the reference group (25-44 years). This finding is similar to the findings in another reported study (22). It has been suggested that children are at a higher risk of AGE due to poor hygienic practices (23). This is supported by the fact that, globally, nearly every child has been infected with AGE at least once by the age of 5 (24).

Higher infection rates may also result from poor immunological status among under-five children.

Seasonal differences in the prevalence of AGE were observed. The prevalence was consistently higher during the high season in all but one of the sentinel sites, with the prevalence ratio ranging from 1.41 to 7.12. The odds of a person being affected with AGE in the high season (Phase 2) were 2.2 times higher than in the low season (Phase 1). Syndromic surveillance data for Barbados support the seasonal trend observed in this study, i.e. during the same timeframe, surveillance data showed the peak of AGE prevalence in the high season (Phase 2). There is also a wealth of evidence that shows the temporal distribution of AGE is bimodal. Viral gastrointestinal illness has been shown to peak in the winter in temperate climates (25,26). In Barbados, the peak in AGE occurred in the period coinciding with the peak in temperate climates. Possible explanations for this observation may include increased illness due to an influx of foreign travellers. A similar seasonal variation has been observed in Cuba and in Argentina (16,27,28).

The odds of exposure (OR 2.4) among respondents with a monthly household income >BD$ 6,000) were twice the odds of respondents with an income below BD$ 2,500. This result was highly significant (p=0.005). This is surprising and contrary to expectations. However, this may result from behaviours associated with higher socioeconomic status, which increases the risk of AGE. For example, those who are wealthy tend to eat more meals away from home. Further, this finding may also have resulted from reporting bias as those in the higher socioeconomic group also are more likely to be better educated and, as such, perceive and report experiencing AGE.

Additionally, a great proportion of the respondents did not give an answer to this query, and in particular, 28% of the cases did not respond. Other demographic and socioeconomic characteristics, including education and cultural identity, were not found to be associated with an increased risk of AGE.

This study assessed consumption of high-risk foods, the purchasing habits of such foods as well as general hygiene practices from a food safety perspective. Analysis of hygiene practices demonstrated that approximately one out of every four cases did not wash their hands with soap before meals. This practice may have contributed to the spread of AGE. Further research aimed at identifying ‘safe’ and ‘unsafe’ food hygiene practices is needed. The results of such research could provide information on other important behavioural practices. Such information is critical for the development of interventions aimed at increased health literacy regarding food hygiene.

Although 44.3% of the respondents indicated that they believed their AGE was related to food consumption, there was no difference in the consumption of foods, such as raw or undercooked eggs, which are often associated with the occurrence of AGE.

This study also provided an opportunity to evaluate the current foodborne disease surveillance system. Ideally, data collected on AGE should be sensitive, complete, timely, and accurate. Evidence of incomplete laboratory reporting of foodborne diseases was demonstrated. In particular, some cases of viral aetiology were not reported to the Ministry of Health. The reporting of laboratory-confirmed cases was also untimely, particularly the reporting of viral pathogens.

Reporting of the norovirus was likely to have been negatively impacted upon by the lack of testing kits. Of equal importance, 57%) of the samples submitted to the laboratory were non-diarrhoeal. This has a negative impact on the laboratory's ability to provide a more accurate descriptive report on the burden and epidemiology of FBD in Barbados. This study, in essence, provided evidence that the attributes of the surveillance system, such as data quality, timeliness, and representativeness, need to be strengthened.

**Figure 8.**
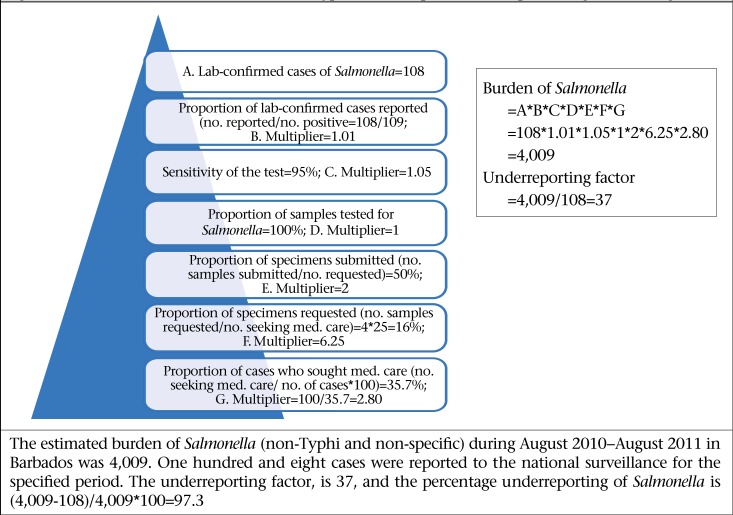
Estimated burden for *Salmonella* (non-Typhi and non-specific) for the period August 2010–August 2011

**Figure 9.**
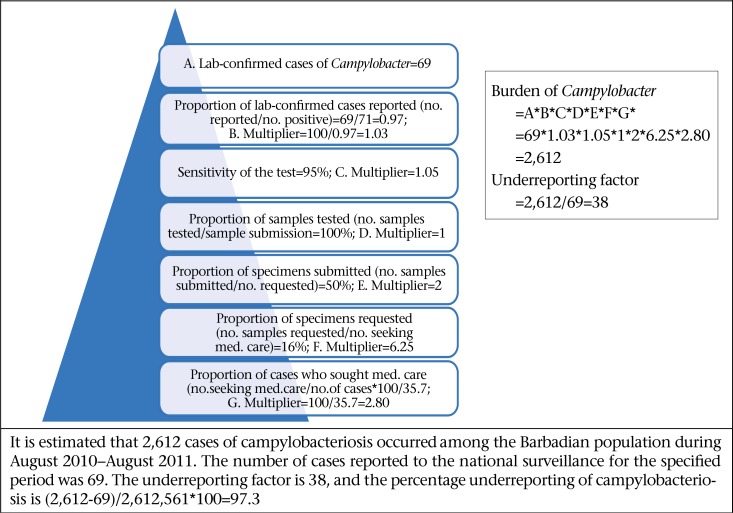
Estimated burden for campylobacteriosis for the period August 2010–August 2011

**Figure 10.**
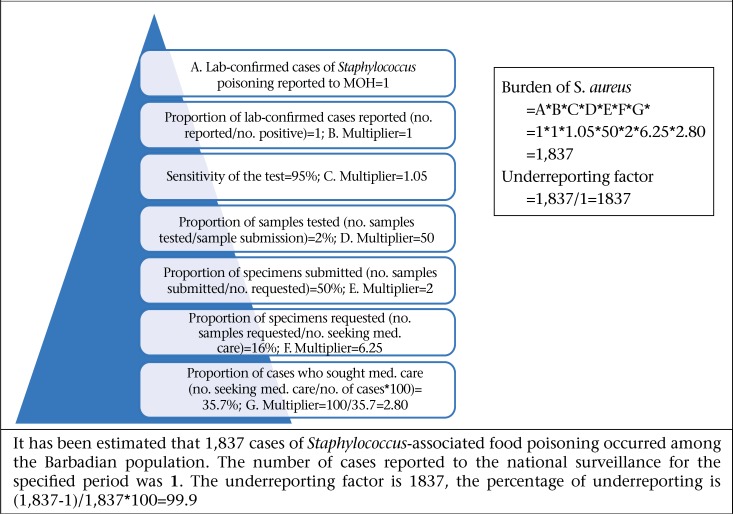
Estimated burden for Staphylococcus aureus during August 2010–August 2011

**Figure 11.**
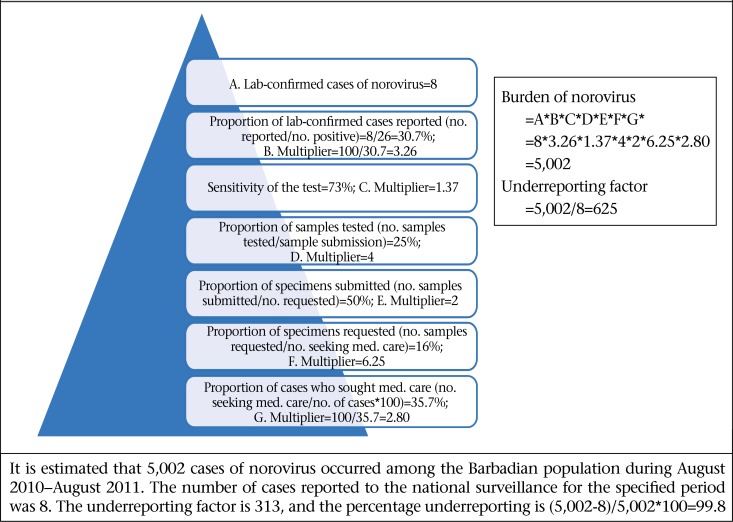
Estimated burden for norovirus for the period August 2010–August 2011

Bacterial pathogens were thought to be the primary aetiologic cause of AGE and FBD in Barbados prior to undertaking this study. This study provides empirical evidence of the epidemiology of FBD in Barbados, and results have shown that viral pathogens, specifically norovirus, are the leading causes of AGE (Figure 8-11). This mirrors the emerging global picture of a changing epidemiology of foodborne diseases with viruses, in particular the norovirus, becoming the leading foodborne pathogen. This changing epidemiology of foodborne illness has been attributed to the increase of ready-to-eat foods, with which the norovirus is associated. Further, the Food and Agriculture Organization have asserted that norovirus and hepatitis are the most common causes of foodborne diseases in developed countries and that they are linked to contamination of fresh produce, seafood, and ready-to-eat foods (29).

### Limitations

The findings of this study should be interpreted in light of the following limitations: the literature shows that retrospective studies of self-reported acute gastrointestinal illness can be subject to recall bias and an overestimation of prevalence (24,30,31). Although the high response rate contributes to the representativeness of the study, selection bias due to differences in the distribution of age, gender, education level, and household income among respondents may be another limitation of this study.

Given that the cases were self-reported, misclassification bias and social desirability bias are also potential limitations of this study because certain responses may have been over- or underreported due to perceived pressure to give the ‘right answer’ (30).

### Conclusions

This study has provided population-based estimates of the public-health burden of AGE and FBD in Barbados. The study has also revealed that acute gastrointestinal illness and foodborne diseases represent a considerable public-health burden in Barbados. These findings also suggest that there is a strong need for behavioural change through increased health literacy regarding food safety to prevent and reduce the burden of AGE and FBD in Barbados. Expansion of the current prevention and education campaigns to include the impact associated with high-risk foods (ready-to-eat foods) could be beneficial in reducing FBD in the population. The necessity for ill persons to submit a diarrhoeal stool sample within 1-2 day(s) of illness onset must also be emphasized. It is also critical that pathogen-based strategies in controlling the spread of AGE are developed. Laboratory surveillance could also benefit from the development of Standard Operating Procedures for reporting and analysis.

Further research is needed to investigate the knowledge, beliefs, and behaviours of the population as these relate to food safety. Knowledge of the magnitude, distribution, and risk factors associated with foodborne diseases is necessary for reducing its burden.

With this baseline information, interventions, targeted surveillance, and research activities can be developed, monitored, and evaluated. Likewise, this fundamental knowledge informs the international estimates, such as the World Health Organization's Global Burden of Disease Assessments, specific to foodborne diseases and AGE.

## ACKNOWLEDGEMENTS

The authors would like to express gratitude to CAREC/PAHO for providing technical guidance and funding for this study. Our deepest gratitude is extended to the Barbados Public Health Laboratory, The Barbados Statistical Department, and the interviewers.

## Figures and Tables

**Figure:**
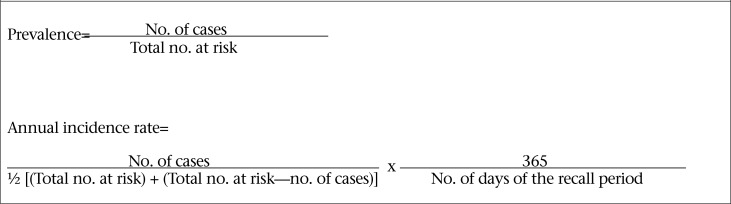
Formulae for calculating prevalence, incidence rate, and incidence proportion
